# Galectin-3 promotes FBXL5-dependent ubiquitination and degradation of YAP1 to constrain colorectal cancer growth

**DOI:** 10.3389/fimmu.2026.1769656

**Published:** 2026-04-21

**Authors:** Xiang Zeng, Tsz Kin Mak, Nan Li, Jia Wang, Zhiliang Huang, Weiqun Lu

**Affiliations:** 1 Department of Gastrointestinal Tumor Surgery, Guangzhou Institute of Cancer Research, the Affiliated Cancer Hospital, Guangzhou Medical University, Guangzhou, China; 2 Department of Internal Medicine, Guangzhou Institute of Cancer Research, The Affiliated Cancer Hospital, Guangzhou Medical University, Guangzhou, China

**Keywords:** colorectal cancer, FBXL5, galectin-3, ubiquitination, YAP1

## Abstract

**Introduction:**

The molecular heterogeneity of colorectal cancer (CRC) profoundly shapes clinical outcomes and treatment response, underscoring the need to elucidate the underlying mechanisms to refine diagnosis and therapy. Although LGALS3, which encodes Galectin-3 (Gal-3), has been implicated in tumor biology, its precise role and regulatory mechanism in CRC remain incompletely understood. This study aimed to define the clinical relevance and mechanistic function of the Gal-3-related signaling axis in CRC.

**Methods:**

We integrated TCGA and single-cell RNA sequencing data to evaluate the clinical significance of LGALS3 in CRC. Molecular investigations, including co-immunoprecipitation and ubiquitination assays, were performed to examine how Gal-3 regulated FBXL5 and YAP1. Functional effects were further assessed using cell proliferation assays and xenograft mouse models to investigate the biological role of the Gal-3-FBXL5-YAP1 axis.

**Results:**

Gal-3 positively correlated with FBXL5 and bound FBXL5 to enhance its expression. Patients with high LGALS3 and FBXL5 expression exhibited better survival. Mechanistically, FBXL5 promoted YAP1 protein degradation through the ubiquitin pathway without altering YAP1 transcript levels, suggesting a post-transcriptional regulatory mechanism. Gal-3 increased FBXL5 abundance and promoted YAP1 degradation, thereby supporting the existence of a Gal-3-FBXL5-YAP1 regulatory axis. In vivo, LGALS3 knockdown markedly increased tumor volume, whereas FBXL5 overexpression reduced tumor growth. YAP1 protein expression patterns were consistent with these findings. Cell proliferation assays and xenograft experiments further supported an antitumor effect of this signaling axis in CRC.

**Conclusion:**

This study suggests that the Gal-3-FBXL5-YAP1 axis plays an important role in restraining CRC progression, expands the mechanistic understanding of Gal-3-associated tumor suppression, and supports Gal-3 as a potential therapeutic target in CRC. Nevertheless, additional validation using TEAD reporter assays and YAP1 rescue or mutant analyses will be required to further strengthen the causal framework of this axis.

## Introduction

Colorectal cancer (CRC) ranks among the most common cancers globally, and its pronounced molecular heterogeneity undermines treatment effectiveness and contributes to unfavorable clinical outcomes (1–5). Deciphering the molecular basis of CRC is crucial for advancing accurate diagnosis and developing tailored therapeutic strategies.

Galectin-3 (Gal-3), a lectin encoded by *LGALS3*, participates in a broad range of biological processes and has been strongly implicated in cancer development and progression, with particular relevance to immuno-oncology (6, 7). As a multifunctional tumor-associated molecule, Gal-3 has long been recognized as a promising therapeutic target due to its crucial roles both intracellularly and extracellularly (8, 9). Intracellularly, Gal-3 modulates cell proliferation, survival, and gene expression, influencing essential cellular functions (10, 11). Gal-3 also functions extracellularly as a “molecular glue,” facilitating cell–cell and cell–matrix contacts and, in turn, shaping fundamental behaviors such as adhesion and migration (12, 13). These multifaceted functions enable Gal-3 to play a critical role in various physiological and pathological processes, particularly in tumor invasion and metastasis, making it a significant focal point in cancer research.

In CRC, the dual roles of Galectin-3 are particularly evident; its functional behavior is largely dictated by its binding partners and the surrounding molecular milieu, which together determine whether it acts in a tumor-promoting or tumor-suppressive manner. Although Gal-3 has been reported to promote tumor progression in multiple cancer types, its role in colorectal cancer appears context-dependent and remains debated across studies. In particular, the functional consequences of Gal-3 may differ by cellular compartment and by cell type within the tumor microenvironment. On the one hand, as a critical component of immune-suppressive pathways such as the STAT3/Gal-3/LAG3 axis, the inhibition of Gal-3 expression can relieve the functional impairment of CD8^+^ T cells, thereby enhancing anti-tumor immune responses (14). On the other hand, recent studies show that periplocin stabilizes and harnesses LGALS3 to drive TRIM16- and ESCRT-dependent mycophagy, exacerbating lysosomal dysfunction and inducing death of CRC cells, which supports an antitumor role for LGALS3 in CRC cells (15). Periplocin binds Gal-3 and, by blocking its ubiquitin-mediated degradation, triggers lethal lysophagy. In line with this mechanism, the function of LGALS3 generally depends on its interactions with partner proteins and on ubiquitin-dependent regulation (16, 17). In the molecular mechanisms of CRC, YAP1 (Yes-associated protein 1), a key effector of the Hippo signaling pathway, has garnered significant attention. Persistent upregulation of YAP1 is tightly linked to malignant tumor progression and drives core cellular programs, including proliferation, survival, and migration (18). Invasive and migratory traits may additionally be shaped by non-genetic cellular state features that are emerging as actionable vulnerabilities in solid tumors (19). Consequently, the ubiquitin-mediated degradation of YAP1 emerges as a crucial mechanism for controlling its activity and stability and is regarded as a significant pathway in suppressing tumor initiation and progression (20). It is well-established that the ubiquitination of YAP1 is regulated by various E3 ubiquitin ligases, including TRIM32, Mdm2, and FBXL5, which, through direct interaction with YAP1, mark it for degradation (21–23). This process helps limit YAP1 accumulation within the cell, preventing its excessive activation, and thereby inhibiting tumor progression. While the ubiquitin regulation of YAP1 has been extensively studied across various tumor types, the precise role of the interaction between Gal-3 and YAP1 in CRC remains inadequately explored. Investigating how Gal-3 may modulate YAP1’s stability and function through ubiquitination could provide a promising new avenue for targeted therapeutic strategies in CRC.

In this study, we primarily focus on tumor cell–intrinsic Galectin-3 and investigate a mechanistic axis linking Gal-3 to FBXL5-dependent ubiquitination and degradation of YAP1. We integrated public datasets and CRC single-cell transcriptomic profiles with *in vitro* assays and xenograft models to interrogate the tumor cell–intrinsic function of Gal-3 in CRC. Across multiple datasets, LGALS3 expression tends to be lower in tumor tissues than in non-tumor controls, and our functional experiments support a model in which Gal-3 attenuates CRC growth phenotypes in the experimental settings examined. Mechanistically, we identify a Gal-3–FBXL5–YAP1 regulatory axis, whereby Gal-3 promotes FBXL5-dependent ubiquitination and degradation of YAP1.Looking forward, large online biobanks and public repositories, facilitated by user-friendly cohort extraction pipelines, will enable broader external validation and subgroup analyses of the Gal-3–FBXL5–YAP1 axis (24). In parallel, AI-driven multi-omics integration is expected to accelerate target prioritization and precision therapy design, supporting more forward-looking translational strategies (25–27).

## Methods

### Sample collection

The study timeframe spanned from October 2021 to July 2025. Guided by the Declaration of Helsinki, the research protocol was officially authorized by the Ethics Committee of the Affiliated Cancer Hospital of Guangzhou Medical University (No. 2020-SZ08). Inclusion criteria were pathologically confirmed colorectal adenocarcinoma, availability of matched tumor and adjacent non-tumor tissues, and either no prior anti-tumor treatment before tissue collection or, when applicable, treatment status recorded as indicated; exclusion criteria included a history of other malignancies, insufficient tissue quantity or quality for molecular assays, and incomplete clinicopathological information. Detailed clinicopathological data for all patients are available in the **Supplementary Table 1**.

### Public data collection and analysis

Datasets were downloaded from the Gene Expression Omnibus (GEO; http://www.ncbi.nlm.nih.gov/geo) and The Cancer Genome Atlas (TCGA; http://gdc.cancer.gov/). We integrated four colorectal cancer single-cell RNA-seq datasets (GSE132465, GSE144735, GSE166555, and GSE178341) containing tumor and normal tissues with available T-stage information. Single-cell processing and downstream analyses were performed in Python using Scanpy. Cells with <200 detected genes or >20% mitochondrial reads were excluded, and doublets were removed with Scrublet. After normalization and log transformation, highly variable genes were identified using Scanpy’s highly_variable_genes function, followed by principal component analysis (PCA). Batch effects were then corrected in PCA space using Harmony prior to unsupervised clustering.

### Oncogene annotation

A curated cancer gene list was retrieved from the OncoKB database (Precision Oncology Knowledge Base). Genes explicitly annotated as Oncogene under the Gene Type category were extracted and used for downstream correlation analysis in cancer cells. The complete list of included genes is provided in **Supplementary Table 2**.

### Survival analysis

For continuous variables, threshold determination was performed in R using surv_cutpoint from the survminer package (v0.4.9), which implements maximally selected rank statistics to define the optimal split point. This method applies the maximally selected rank statistics approach to determine the threshold that best separates patients into groups with distinct survival outcomes. Patients were subsequently categorized into high-expression and low-expression groups based on these optimal cutoffs.

Survival outcomes were evaluated using Kaplan–Meier (KM) estimates and univariate Cox proportional hazards models in the R survival package (v3.5.7). KM estimates were used to visualize survival patterns across groups, and group separation was evaluated with the log-rank test. In parallel, gene expression was entered into univariate Cox proportional hazards models to obtain effect sizes as HRs with 95% CIs for overall survival.

### Differential gene ranking and GO enrichment analysis

For bulk RNA-seq data, we conducted differential expression testing in R (v4.3.0) with the limma package (v3.56.2). DEGs were obtained using the topTable function, and genes meeting the thresholds of adjusted P < 0.01 and |log2FC| > 0.35 were defined as significant.

For single-cell RNA-seq, the Scanpy package (version 1.9.6) in Python was used. The rank_gene_groups function, based on the Wilcoxon rank-sum test, was applied to identify DEGs across clusters. A volcano plot was generated using Matplotlib (version 3.5.2) to visualize the results, and the top seven upregulated and bottom seven downregulated marker genes were selected. Their expression patterns across clusters were displayed using a dot plot (scanpy.pl.dotplot). Subsequently, functional enrichment analysis of DEGs from both bulk and single-cell datasets was performed using Metascape (https://metascape.org) to identify significantly enriched Gene Ontology (GO) terms and biological pathways.

### Molecular docking

Protein structures of LGALS3, FBXL5, and YAP1 were downloaded from the RCSB Protein Data Bank (PDB; http://www.rcsb.org/). Specifically, the carbohydrate recognition domain (CRD) of LGALS3 (PDB ID: 2XG3; residues 114–250), the LRR-containing region of FBXL5 (PDB ID: 6VCD; chain B; residues 183–674), and the first WW domain of human YAP1 (PDB ID: 4REX; residues 165–209) were used. For 6VCD, only chain B corresponding to FBXL5 was retained, and other chains were removed prior to docking. Crystallographic water molecules and small ligands were removed, and missing atoms were repaired before simulations. Docking simulations were carried out using MegaDock-GPU (v4.1.3) with default scoring parameters. The predicted interaction interfaces were visualized using PyMOL.

### Multivariable Cox proportional hazards regression analysis

To further evaluate the independent prognostic value of LGALS3 and FBXL5, multivariable Cox proportional hazards regression analyses were performed. Overall survival (OS) was used as the primary endpoint. Variables included in the multivariable models comprised age, sex, AJCC stage, and gene expression group (high vs. low). Hazard ratios (HRs) with 95% confidence intervals (CIs) were calculated. The proportional hazards assumption was assessed using Schoenfeld residuals. All survival analyses were conducted using the lifelines (v0.27.8) package in Python.

### Cell culture and transfection

SW480 and HCT116 cells were purchased from Procell (Wuhan, China) and maintained in DMEM containing 10% fetal bovine serum and 1% penicillin–streptomycin at 37 °C in a humidified incubator with 5% CO_2_. Cells were routinely tested and confirmed to be mycoplasma-free. Transient transfection was carried out with Lipofectamine™ 3000 following the manufacturer’s instructions. In brief, 2 µg plasmid DNA and 5 µL Lipofectamine™ 3000 were diluted separately in 200 µL Opti-MEM, mixed. Six hours post-transfection, the transfection mixture was removed and replaced with fresh complete medium. A description of the plasmids is included in **Supplementary Table 3**.

### RNA extraction and RT-qPCR analysis

Total RNA was extracted from frozen tissue samples using TRIzol reagent (Invitrogen) and reverse transcribed into cDNA with the cDNA Reverse Transcription Kit (Applied Biosystems, 4368814). Quantitative PCR was performed using the KAPA SYBR FAST qPCR Kit (Roche, 07959567001) according to the manufacturer’s instructions. The primers used are listed in **Supplementary Table 4**.

### Western blot

After lysis, protein samples were quantified, separated by SDS–PAGE, and transferred onto PVDF membranes. Membranes were blocked with 5% BSA for 2 h, incubated with primary antibodies overnight, then with secondary antibodies for 2 h, followed by signal detection using ECL. Antibodies are listed in **Supplementary Table 5**.

### Cell viability experiment

A total of 5 × 10^3 cells were dispensed into each well of a 96-well plate and maintained for 24 h. Subsequently, 10 μL of CCK-8 reagent (Beyotime, C0038-500) was added to each well and incubated for 2 h. The optical density was then recorded at 450 nm using a microplate reader. Cell viability was determined from the absorbance values, and cell numbers were estimated based on a standard curve.

### Colony formation assay

After transfection, logarithmic-phase cells were seeded at 1000 cells per well in 6-well plates and cultured for 7 days with medium changes every two days. Colonies were fixed with 4% PFA, stained with 0.5% crystal violet, and counted under a microscope. All experiments were performed in triplicate.

### EdU assay

After transfection or drug exposure, cells were cultured to ~80% confluence and incubated with 10 μM EdU (Beyotime) for 2 h. Cells were then fixed with 4% paraformaldehyde, permeabilized using 0.5% Triton X-100, and subjected to Click-iT detection according to the manufacturer’s protocol. Nuclei were counterstained with DAPI, and EdU-positive cells were imaged and quantified by fluorescence microscopy to evaluate proliferation.

### Ubiquitination assay

To assess the ubiquitination level of YAP1, SW480 cells from the shFBXL5 group and shNC group were treated with 10 μM MG132 (Med Chem Express, HY-13259) for 6 h to block proteasomal degradation. Cells were lysed using pre-chilled RIPA buffer containing protease inhibitors. After quantification of the lysates, equal amounts of total protein were incubated with anti-YAP1 antibody at 4 °C for 2–4 h. Immune complexes were captured with Protein A/G agarose beads overnight at 4 °C. Following extensive washing with PBS, the bound proteins were eluted and analyzed by SDS-PAGE and western blotting. Ubiquitinated YAP1 was detected using an anti-ubiquitin antibody, with Input samples serving as loading controls.

### Protein stability and degradation experiment

Protein half-life was examined in SW480 cells overexpressing FBXL5 and in matched control cells using a cycloheximide (CHX) chase. Cells were exposed to CHX (100 μg/mL; MedChemExpress, HY-12320) and harvested at 0, 4, 6, 8, and 12 h for protein extraction, followed by Western blotting. To evaluate proteasome-dependent degradation, FBXL5-overexpressing and control cells were incubated with MG132 (10 μM) for 6 h, with vehicle (DMSO) used as the control. Whole-cell lysates were then collected for Western blot analysis.

### Animal experiments

Six-week-old BALB/c nude mice (n = 24; Cavens Laboratory Animal Co., Ltd., Changzhou, China) were maintained under SPF conditions (20 ± 2 °C) with ad libitum access to food and water. Animals were randomly allocated into four groups (n = 6 per group) and baseline body weights were checked to ensure balance across groups before inoculation. Each mouse received a subcutaneous injection of 3 × 10^6^ stably transfected SW480 cells. Tumor dimensions were recorded daily using calipers to measure length (L) and width (W), and tumor volume was calculated as (L × W^2^)/2. To reduce bias, cages were coded and tumor measurements and data analysis were performed by an investigator unaware of group allocation. Humane endpoints were predefined; mice were euthanized if tumor ulceration occurred, if body weight loss exceeded 20%, if animals showed signs of distress, or if tumor volume exceeded 1,500 mm^3^. On day 21, mice were deeply anesthetized with inhaled isoflurane (R510-22-10; RWD, Shenzhen, China) at 4% with an oxygen flow rate of 2 L/min and subsequently euthanized by cervical dislocation as a secondary physical method to ensure death, according to the protocol approved by the Experimental Animal Ethics Committee of Guangzhou Medical University (No. S2020-100). The study is reported in accordance with ARRIVE guidelines.

### Statistical analysis

Continuous variables were tested for distributional assumptions prior to hypothesis testing. Unless otherwise stated, n denotes the number of independent biological replicates (independent experiments or independent biological samples); technical replicates, when performed, were averaged within each biological replicate and were not counted as n. For comparisons between two independent groups, a two-sided Student’s t-test was used when assumptions were satisfied; otherwise, a two-sided Wilcoxon rank-sum test was applied. For experiments involving three or more groups, one-way ANOVA was performed followed by Holm-adjusted *post hoc* pairwise comparisons; when parametric assumptions were not met, the Kruskal–Wallis test was used followed by Holm-adjusted pairwise Wilcoxon rank-sum tests. For time-course assays, group-by-time effects were evaluated using two-way ANOVA or mixed-effects models when appropriate, with Holm-adjusted *post hoc* comparisons across time points. Effect sizes are reported as mean differences with 95% confidence intervals for parametric analyses and as rank-biserial correlation for nonparametric two-group comparisons. Data are presented as mean ± SEM unless otherwise stated; the definition of error bars and the number of independent replicates for Western blot densitometry are specified in the corresponding figure legends. A two-sided *P* value < 0.05 was considered statistically significant. Most statistical analyses and visualizations were performed in Python (version 3.8) using SciPy (version 1.10.1) and Matplotlib (version 3.5.2); survival analyses were performed as specified in the relevant Methods subsection.

## Results

### Downregulated *LGALS3* in CRC, with higher expression indicating better prognosis

**Figure 1:**
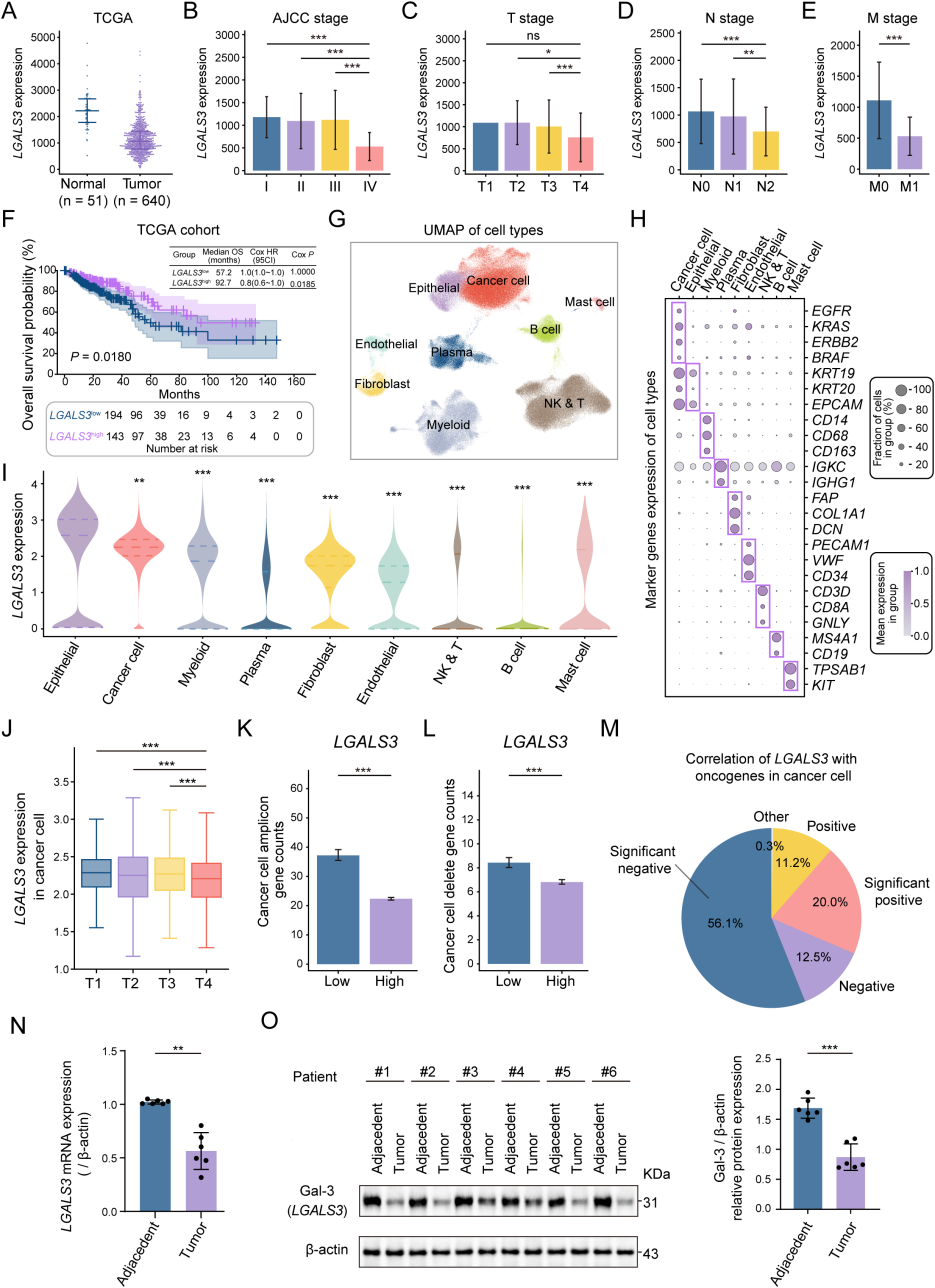
Expression differences of LGALS3 gene and protein between tumor and normal tissues. **(A)** *LGALS3* expression in normal (n=51) and tumor (n=640) tissues from TCGA. **(B-E)** Expression of *LGALS3* across different AJCC stages, T, N, and M stages in the GEO dataset. **(F)** Kaplan–Meier analysis of overall survival in TCGA CRC cohort grouped by LGALS3 expression levels. Patients were stratified into low-expression (n = 194) and high-expression (n = 143) groups, *P* = 0.018. **(G)** UMAP plot of the 9 major cell types derived from integrating four single-cell datasets of CRC patients. **(H)** Expression levels of marker genes for each cell type, where darker colors indicate higher average expression levels and larger dots represent a higher proportion of the gene in the cell. **(I)** Expression levels of *LGALS*3 in different cell types. **(J)** Expression of *LGALS3* in cancer cells. **(K)** Gene amplification counts in cancer cells with high and low *LGALS3* expression. **(L)** Gene deletion counts in cancer cells with high and low *LGALS3* expression. **(M)** Correlation of *LGALS*3 with oncogenes in cancer cells, with a significant negative correlation observed in 56.1% of genes. **(N)** mRNA expression levels of *LGALS3* in tumor and adjacent normal tissues from cancer patients, n = 6. **(O)** Protein levels of Gal-3 in tumor and adjacent normal tissues from cancer patients, n = 6. Ns, *P* > 0.05; *, *P* < 0.05; **, *P* < 0.01; ***, *P* < 0.001.

TCGA-based analyses revealed that *LGALS3* expression was enriched in normal colorectal tissues relative to tumors and declined stepwise with increasing disease burden, including higher AJCC stage and more advanced T, N, and M classifications. (**Figure 1A**, **Supplementary Figures 1A–D**). Similarly, the GEO dataset showed a consistent decrease in *LGALS3* expression across advancing AJCC, T, N, and M stages, with statistically significant differences (**Figures 1B–E**). More importantly from a clinical perspective, low *LGALS3* expression was associated with poor patient survival (**Figure 1F**). In line with this, multivariable Cox regression further identified high *LGALS3* expression as an independent favorable prognostic factor after adjusting for age, sex, and AJCC stage (**Supplementary Figure 1F**). Next, we integrated scRNA-seq data from four CRC patient datasets, resulting in 529920 cells. After filtering out low-quality cells and doublets, 284,932 cells were retained for downstream analysis (**Supplementary Figure 1E**). Through this process, nine primary cell clusters were classified by their unique gene expression profiles with high expression, including cancer cells, epithelial, myeloid, plasma cell, fibroblast, endothelial, NK and T cell, B cell, and mast cell (**Figures 1G, H**). Unexpectedly, *LGALS3* was found to be much more abundant in non-malignant epithelial cells than in cancer cells and other cell types (**Figure 1I**). Furthermore, the expression of *LGALS3* in cancer cells progressively decreased during tumor progression, a finding consistent with the results from public datasets (**Figure 1J**).

Additionally, cancer cells with low LGALS3 expression exhibited significantly higher amplification and deletion counts than those with high LGALS3 expression, indicating an increased copy-number alteration burden and a more genomically unstable malignant state (**Figures 1K, L**). Notably, LGALS3 also showed significant inverse associations with more than half of the oncogenes analyzed, further supporting its potential tumor-suppressive role in CRC (**Figure 1M**). Based on preliminary exploration, qPCR results showed that *LGALS3* is indeed highly expressed in normal tissues compared to tumor tissues (**Figure 1N**). Meanwhile, the protein levels of Gal-3 were also significantly higher in normal tissues than in tumor tissues (**Figure 1O**). In conclusion, we found that the low expression of *LGALS3* and Gal-3 in CRC was closely associated with tumor progression, and their expression may be crucial in preventing tumor advancement.

### *LGALS3* restrained malignant phenotypes of CRC cells

**Figure 2:**
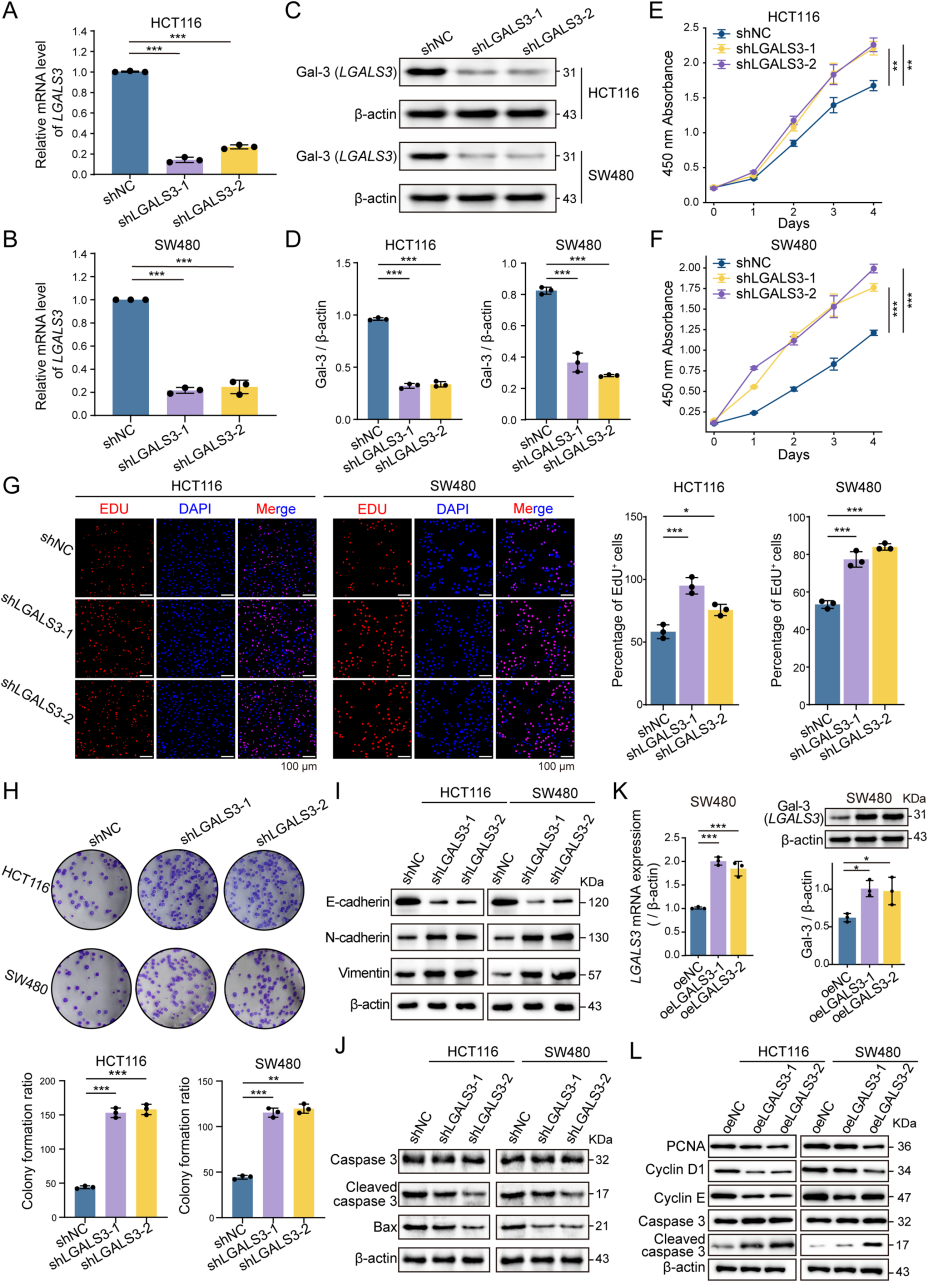
Knockdown of LGALS3 promotes the growth of HCT116 and SW480 cells. **(A, B)** mRNA levels of *LGALS3* after knockdown in HCT116 and SW480 cells, n = 3. **(C, D)** Protein levels of Gal-3 after knockdown in HCT116 and SW480 cells, n = 3. **(E, F)** Cell proliferation ability after LGALS3 knockdown assessed by CCK-8 assay, n = 3. **(G)** EdU staining to assess cell proliferation after LGALS3 knockdown, n = 3. **(H)** Effect of LGALS3 knockdown on colony formation ability of HCT116 and SW480 cells, n = 3. **(I)** Knockdown of LGALS3 resulted in decreased expression of E-cadherin and increased expression of N-cadherin and Vimentin in tumor cells, n = 3. **(J)** Knockdown of LGALS3 reduces the expression of apoptosis-related proteins in cancer cells, n = 3. **(K)** Overexpression of *LGALS3* in SW480 cells was validated by increased *LGALS3* mRNA levels and elevated Gal-3 protein expression, n = 3. **(L)** Overexpression of *LGALS3* led to decreased expression of the proliferation markers PCNA, Cyclin D1, and Cyclin E, and increased expressions of Cleaved caspase-3 in HCT116 and SW480 cells, n = 3. *, *P* < 0.05; **, *P* < 0.01; ***, *P* < 0.001.

We next investigated the tumor-suppressive potential of *LGALS3 in vitro*. shRNA-mediated knockdown effectively reduced *LGALS3* expression at both the mRNA (**Figures 2A, B**) and protein level (**Figures 2C, D**). The absence of *LGALS3* significantly enhanced the proliferative capacity of both HCT116 and SW480 colon cancer cell lines. CCK8 assay results showed that from days 2 to 4, cell proliferation was markedly higher in the shLGALS3 group compared with the control (shNC), and cell viability was significantly increased on day 4 (**Figures 2E, F**). Furthermore, EdU staining confirmed that LGALS3 knockdown significantly increased the percentage of cells in the proliferative phase (**Figure 2G**). Colony formation assays further corroborated this growth-promoting effect, with the shLGALS3 group producing more and larger colonies (**Figure 2H**). Furthermore, *LGALS3* ablation was accompanied by molecular changes consistent with a more aggressive phenotype, including altered EMT-related protein expression and reduced apoptotic markers in CRC cells (**Figures 2I, J**). In addition, upon *LGALS3* overexpression, we observed molecular changes consistent with a growth-suppressive role: reduced levels of the proliferation markers PCNA and Cyclin D1 and increased apoptotic activation, as indicated by elevated cleaved caspase-3 (**Figures 2K, L**), providing additional molecular evidence that *LGALS3* restrains CRC cell growth.

### *LGALS3* positively regulated *FBXL5*, and high *FBXL5 e*xpression was associated with improved survival

**Figure 3:**
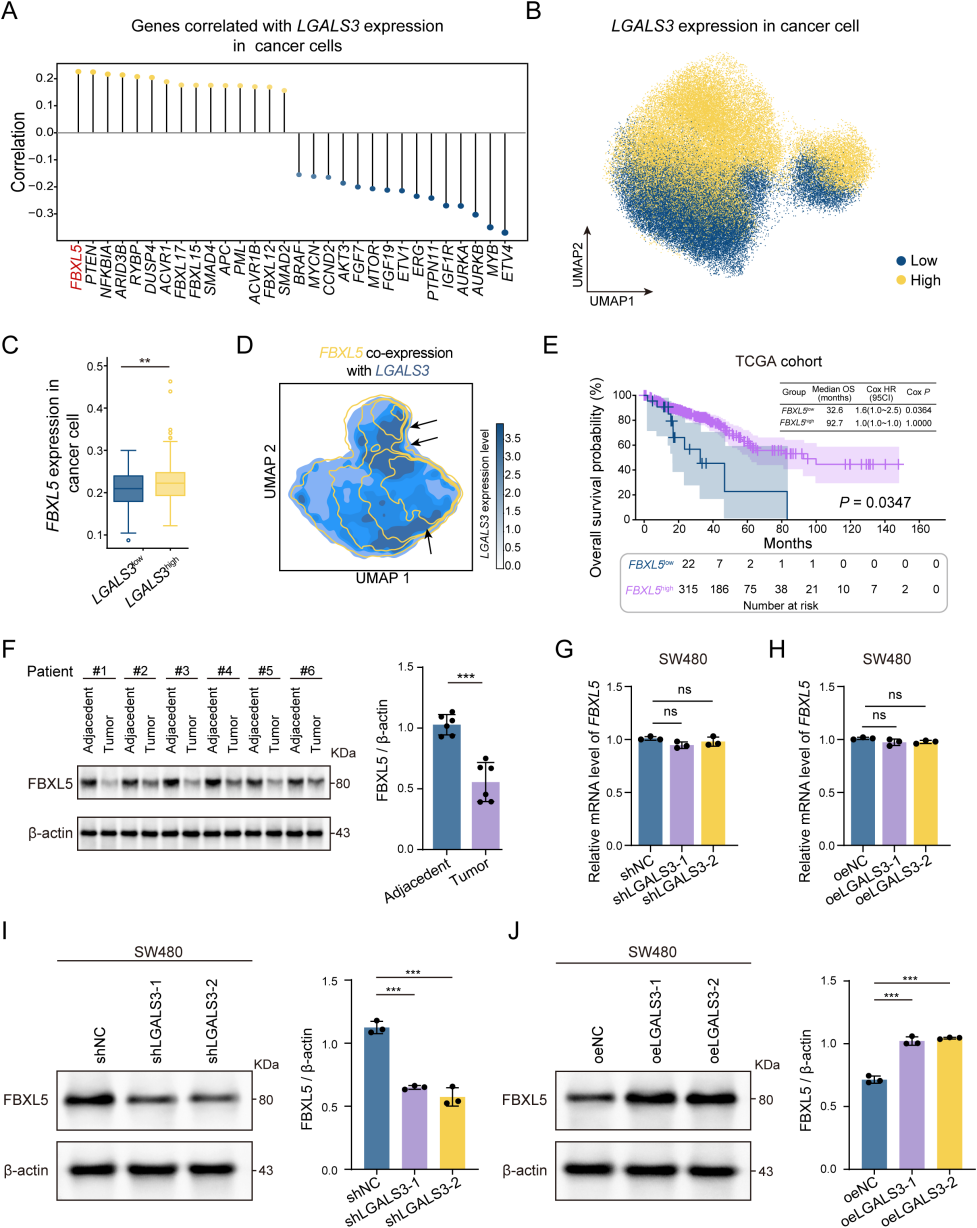
LGALS3 regulates FBXL5 expression. **(A)** Analysis of *LGALS3*-correlated genes based on single-cell transcriptomic data, showing the top 15 positively (yellow) and negatively **(blue)** correlated genes. **(B)** UMAP plot showing LGALS3-high and LGALS3-low cancer cells, defined according to the median normalized LGALS3 expression level, with low-expression cells shown in blue and high-expression cells shown in yellow. **(C)** *FBXL5* expression is significantly higher in cancer cells with high *LGALS3* expression. **(D)** Co-expression contour plot indicates spatial colocalization of *FBXL5* and *LGALS3* in UMAP space. **(E)** Kaplan–Meier analysis of overall survival in the TCGA CRC cohort grouped by *FBXL5* expression levels; patients are stratified into low-expression (n = 22) and high-expression (n = 315) groups, *P* = 0.0347. **(F)** Paired tissue analysis from CRC patients shows lower FBXL5 protein levels in tumor tissues compared to adjacent normal tissues, n = 6. **(G, H)** Neither knockdown nor overexpression of *LGALS3* significantly altered *FBXL5* mRNA levels in SW480 cells, n = 3. **(J)** Knockdown of LGALS3 significantly reduces FBXL5 protein expression, n = 3. **(K)** Overexpression of LGALS3 significantly increases FBXL5 protein expression, n = 3. *, *P* < 0.05; **, *P* < 0.01; ***, *P* < 0.001. .

Building on the phenotypic observations above, we next investigated the tumor-suppressive mechanism of *LGALS3*. At the level of cancer cells, scRNA-seq analysis systematically profiled genes whose expression correlates with *LGALS3*, and we present the top 15 positive and top 15 negative correlates (**Figure 3A**). Notably, the positively correlated set was enriched for canonical tumor-suppressive nodes such as *PTEN*, *RYBP*, and *APC*, indicating that higher *LGALS3* aligns with an anti-proliferative molecular program. Among these, *FBXL5*, a subunit of the SCF E3 ubiquitin ligase complex, stood out by correlation strength and biological plausibility, and was prioritized as a candidate mediator. Cancer cells were stratified into *LGALS3*-high and *LGALS3*-low groups according to the median normalized *LGALS3* expression level, yielding 38,125 cells in each group. *FBXL5* was preferentially enriched in the *LGALS3*-high compartment, and UMAP mapping showed substantial overlap between the two features, suggesting coordinated expression (**Figures 3B–D**). In CRC patients, the TCGA cohort showed that higher *FBXL5* expression was associated with superior overall survival, consistent with a tumor-suppressive role (**Figure 3E**). Multivariate Cox regression analysis further confirmed that higher FBXL5 expression remained an independent favorable prognostic factor after adjusting for age, sex, and AJCC stage (**Supplementary Figure 2A**). Concordantly, paired clinical specimens showed that FBXL5 protein levels were reduced in tumor tissues compared with adjacent non-tumor tissues (**Figure 3F**). In cell-based assays, modulation of LGALS3 did not significantly alter *FBXL5* mRNA levels in SW480 cells, as neither LGALS3 knockdown nor overexpression changed *FBXL5* transcript abundance (**Figures 3G, H**). By contrast, FBXL5 protein abundance was clearly responsive to *LGALS3* perturbation: silencing *LGALS3* decreased FBXL5 protein levels, whereas *LGALS3* overexpression increased FBXL5 protein levels (**Figures 3I, J**).

Together, these data suggest that the positive association between *LGALS3* and FBXL5 is more prominently manifested at the protein level, consistent with regulation beyond transcriptional control.

### LGALS3 modulated YAP1 protein expression via FBXL5

**Figure 4:**
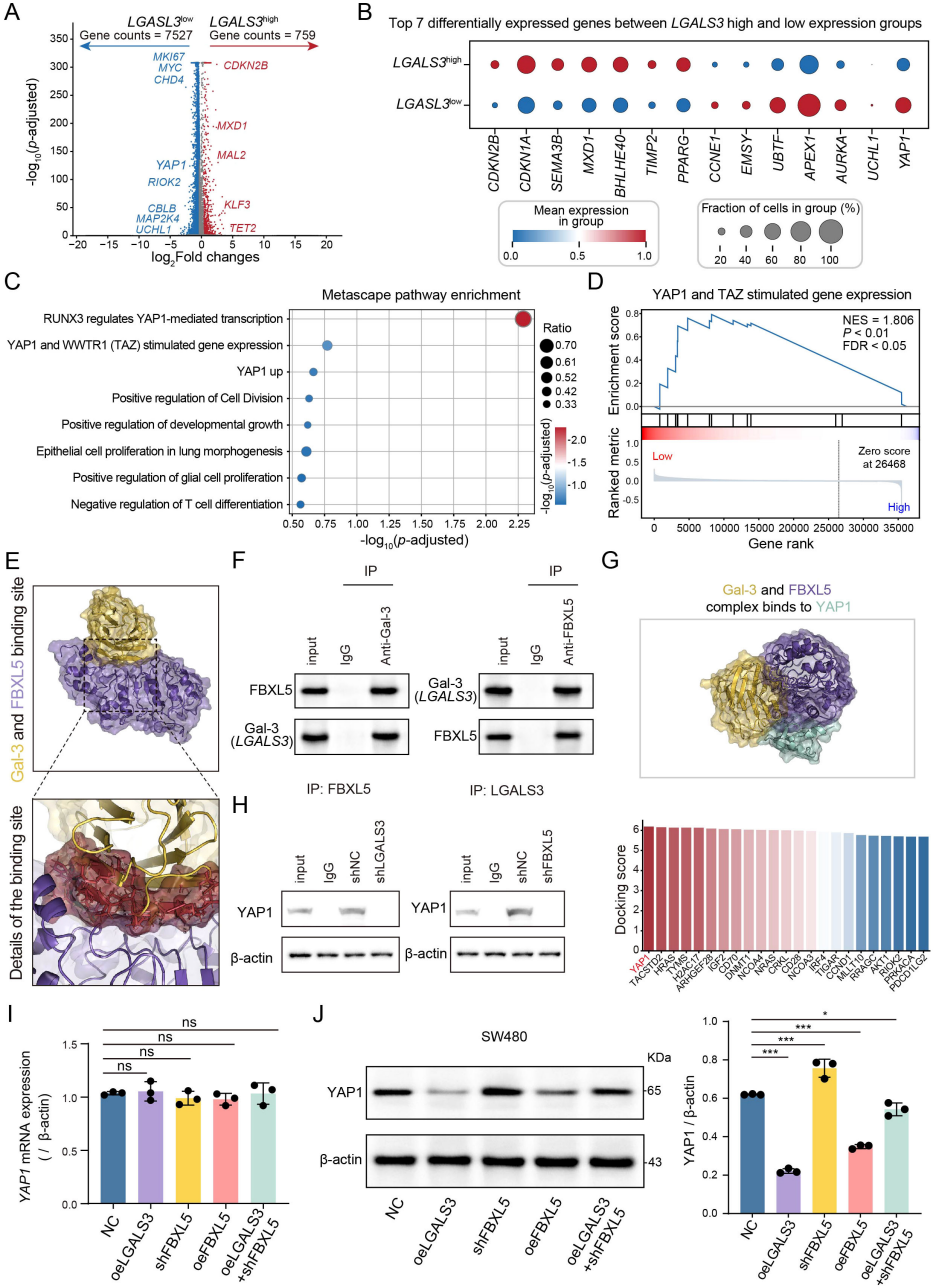
Gal-3 regulates YAP1 activity through FBXL5-mediated interaction. **(A)** Volcano plot of differentially expressed genes between *LGALS3* high and low cancer cell groups. **(B)** Top seven significant genes in *LGALS3* high and low cancer cell groups; the redder the color, the higher the expression level, and the larger the dot, the greater the proportion of cells expressing the gene. **(C)** Bubble plot of pathway enrichment analysis based on genes from the *LGALS3* low-expression group; color represents significance, and dot size indicates the ratio of intersecting genes to total pathway genes. **(D)** Pathway enrichment analysis of genes regulated by YAP1 and TAZ. **(E)** Molecular docking of Gal-3 (yellow) with FBXL5 (purple) and corresponding magnified views. **(F)** Co-IP analysis of Gal-3 and FBXL5. **(G)** Molecular docking model of the Gal-3-FBXL5 complex with YAP1 (top); docking scores of the Gal-3-FBXL5 complex with YAP1 among oncogenic genes regulated by *LGALS3* (bottom). **(H)** Left: FBXL5 was immunoprecipitated and YAP1 binding was assessed upon LGALS3 knockdown. Right: LGALS3 was immunoprecipitated and YAP1 binding was assessed upon FBXL5 knockdown. **(I)** Effect of *LGALS3* and FBXL5 on YAP1 mRNA levels, n = 3. **(J)** Effect of Gal-3 and FBXL5 on YAP1 protein levels, n = 3. ns, *P* > 0.05; *, *P* < 0.05; **, *P* < 0.01; ***, *P* < 0.001.

To fully elucidate *LGALS3*’s tumor-suppressive mechanism, we further investigated the key downstream signaling pathways regulated by the Gal-3-FBXL5 axis. Differential expression analysis revealed marked transcriptional differences between *LGALS3* high and low tumors (**Figure 4A**). Specifically, *LGALS3*-low tumors displayed striking enrichment of well-known oncogenic drivers, including *CCNE1*, *APEX1*, *AURKA*, *UCHL1*, and *YAP1*, underscoring the preferential activation of proliferative and oncogenic programs (28). In contrast, *LGALS3* high tumors exhibited upregulation of *CDKN2B*, *CDKN1A*, *SEMA3B*, *MXD1*, and *BHLHE40*, indicative of cell-cycle inhibition and immune regulatory signaling (**Figure 4B**) (29, 30). Intriguingly, pathway enrichment based on *LGALS3*-low genes converged on the YAP1 signaling axis, showing high activity (**Figure 4C**). GSEA further reinforced this, highlighting significant enrichment of YAP1-related signatures (**Figure 4D**). Collectively, these results suggest that loss of LGALS3 activity channels cancer cells into a YAP1-dependent proliferative state. Thus, we hypothesize that high Gal-3 expression may exert suppression by modulating FBXL5, which affects YAP1 stability.

Considering Gal-3 is known to interact with various proteins, we investigated whether its positive regulation of FBXL5 expression is mediated through direct physical interaction. To explore this, we utilized molecular docking to predict potential binding sites between Gal-3 and FBXL5 (**Figure 4E**; **Supplementary Table 6**). We then validated the interaction experimentally by co-immunoprecipitation, which confirmed that Gal-3 and FBXL5 associate in cells (**Figure 4F**). In parallel, we explored whether Gal-3 might directly engage YAP1 using the same docking framework; under the tested parameters, we did not obtain a stable docking pose supporting a direct Gal-3–YAP1 interface (**Supplementary Figure 2B**). We therefore examined the possibility that YAP1 regulation by Gal-3 occurs indirectly through FBXL5. Docking of a Gal-3–FBXL5 complex against candidate proteins prioritized from expression correlation analyses identified YAP1 as one of the highest-scoring candidates (**Figure 4F**; **Supplementary Table 7**), prompting subsequent experimental validation. Notably, upon knockdown of either FBXL5 or LGALS3, YAP1 co-precipitation with Gal-3 or FBXL5 was no longer detectable (**Figure 4H**).

Consistent with a post-transcriptional mechanism, overexpression of LGALS3 or FBXL5 did not significantly alter YAP1 mRNA levels, whereas YAP1 protein abundance was markedly affected (**Figure 4H**). Specifically, Gal-3 overexpression reduced YAP1 protein levels, while FBXL5 knockdown increased YAP1 abundance. Importantly, FBXL5 depletion abrogated the Gal-3–mediated reduction of YAP1 protein (**Figure 4I****),** supporting that FBXL5 is required for Gal-3–dependent control of YAP1 at the protein level.

### FBXL5 promoted YAP1 ubiquitination and degradation via the proteasome pathway

**Figure 5:**
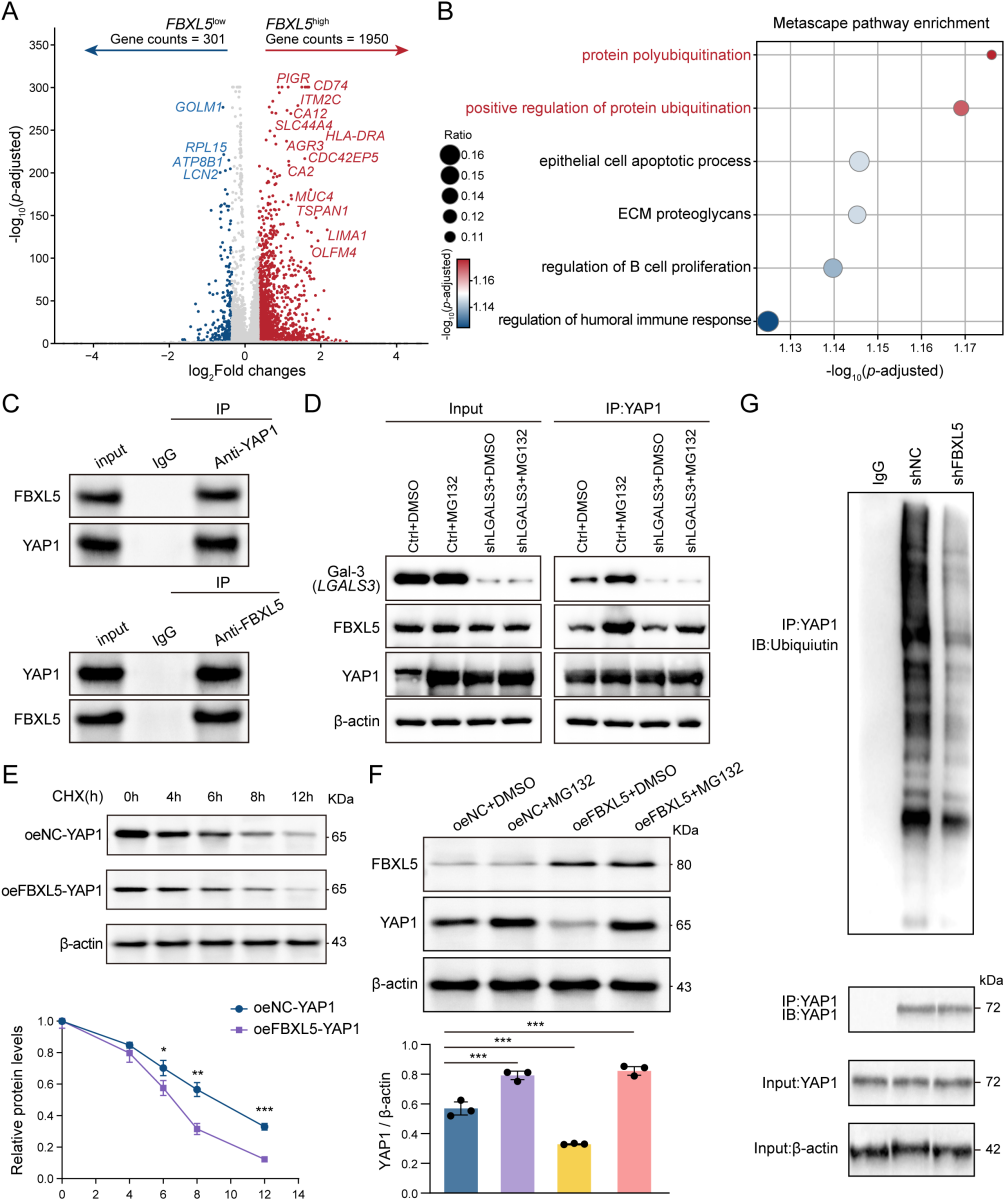
FBXL5-mediated YAP1 ubiquitination and protein degradation. **(A)** Volcano plot showing differentially expressed genes between *FBXL5*-high and *FBXL5*-low expressing cancer cells. **(B)** Metascape pathway enrichment analysis of differentially upregulated genes in the *FBXL5*-high group. The color of the dots represents the significance of the pathway (-log10(p-adjusted)), and the dot size represents the ratio of enriched genes to the total genes in the pathway. **(C)** Co-IP experiments validate the interaction between FBXL5 and YAP1. **(D)** SW480 cells with or without LGALS3 knockdown were treated with DMSO or MG132, followed by immunoprecipitation using an anti-YAP1 antibody and immunoblotting with anti-FBXL5 and anti-YAP1 antibodies. **(E)** YAP1 protein levels in SW480 cells with protein synthesis blocked by cycloheximide (CHX), n = 3. **(F)** YAP1 protein levels in MG132-treated SW480 cells with overexpressed FBXL5 and control groups, n = 3. **(G)** Knockdown of FBXL5 significantly inhibits the polyubiquitination level of YAP1 in SW480 cells. *, *P* < 0.05; **, *P* < 0.01; ***, *P* < 0.001.

It is well-established that FBXL5, a member of the E3 ligase family, is responsible for covalently attaching ubiquitin molecules to target proteins, thereby marking them for proteasomal degradation. Given that the protein levels and function of YAP1, a key oncoprotein, are tightly regulated by the ubiquitination-mediated degradation pathway, we sought to determine whether FBXL5 could degrade YAP1 via this mechanism. Our initial findings from the differential gene expression analysis showed that in cancer cells with high FBXL5 expression, the protein polyubiquitination pathway was significantly responsive, as evidenced by both volcano plot analysis and subsequent pathway enrichment (**Figures 5A, B**). To assess whether FBXL5 physically associates with YAP1 in cells, we performed endogenous co-immunoprecipitation assays. FBXL5 was detected in anti-YAP1 immunoprecipitates, and YAP1 was detected in reciprocal anti-FBXL5 immunoprecipitates (**Figure 5C**), indicating an endogenous FBXL5-YAP1 interaction. Furthermore, knockdown of LGALS3 markedly attenuated the interaction between FBXL5 and YAP1, and treatment with MG132 failed to restore their association. These findings suggest that Gal-3 facilitates the formation or stabilization of the FBXL5–YAP1 complex rather than merely affecting protein stability (**Figure 5D**).

Subsequently, a protein stability assay demonstrated that in cells treated with cycloheximide (CHX), the overexpression of FBXL5 significantly accelerated the degradation rate of the YAP1 protein, markedly shortening its half-life (**Figure 5E**). To determine whether this degradation required the ubiquitin–proteasome system, cells were exposed to the proteasome blocker MG132. As anticipated, the addition of MG132 completely reversed the downregulation of YAP1 protein levels caused by FBXL5 overexpression (**Figure 5F**). Consistent with these findings, knockdown of FBXL5 led to a significant decrease in YAP1’s ubiquitination level (**Figure 5G**). Taken together, these results led us to conclude that FBXL5 can promote YAP1 degradation by interacting with it and facilitating its degradation via the ubiquitin-proteasome pathway.

### Gal-3 exerted its tumor-suppressive role in CRC via the FBXL5-YAP1 axis

**Figure 6:**
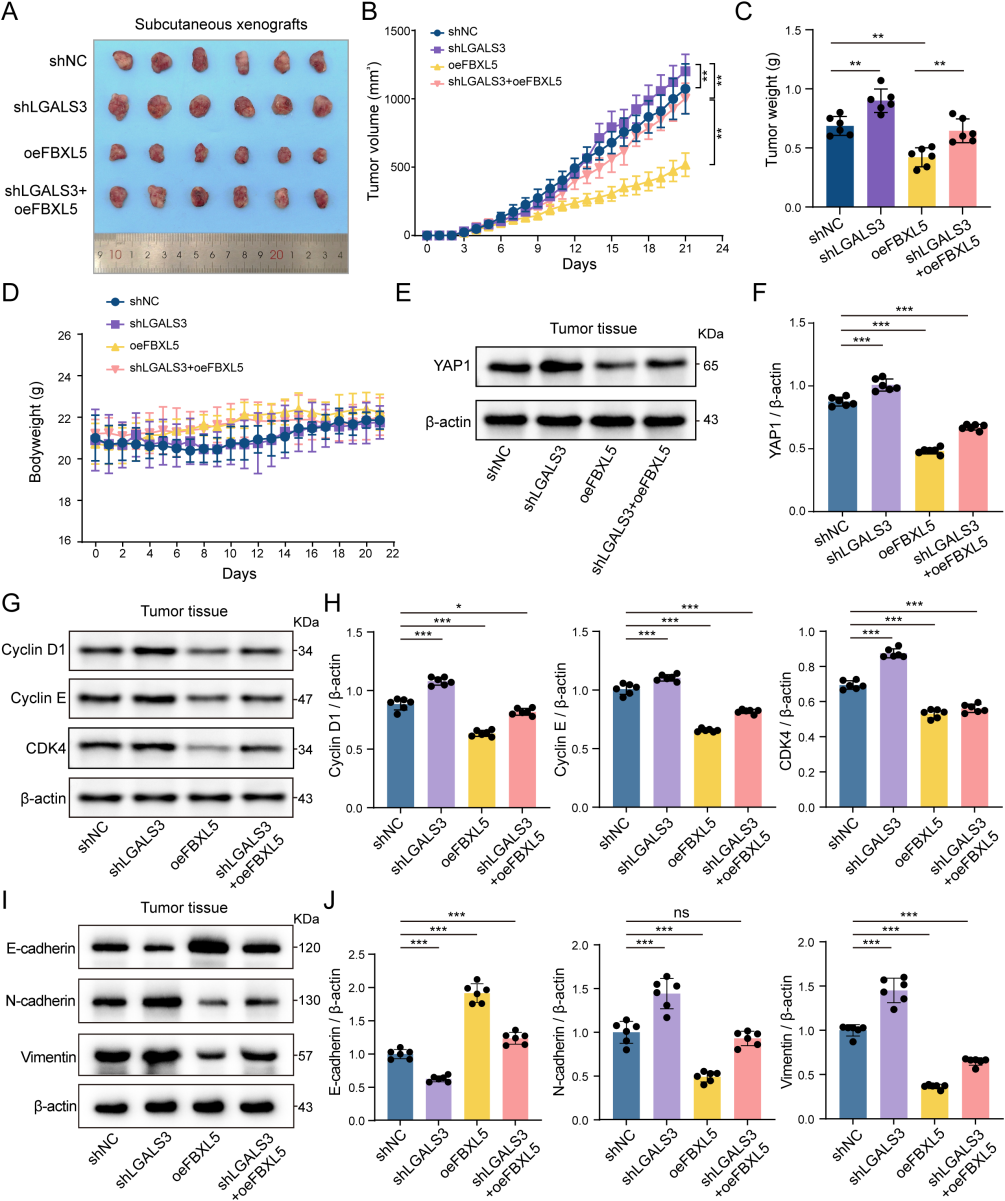
LGALS3 and FBXL5 regulated tumor growth and protein expression in subcutaneous xenografts. **(A)** Representative images of subcutaneous xenograft tumors from each group, n = 6. **(B)** Tumor volume change curves of each group over time, n = 6. **(C)** Final tumor weights of each group at the end of the experiment, n = 6. **(D)** Body weight change curves of tumor-bearing mice, n = 6. **(E)** Western blot analysis of YAP1 protein levels in tumor tissues, n = 6. **(F)** Quantitative analysis of YAP1 protein levels based on Western blot results. **(G,H)** Western blot analysis of Cyclin D1, Cyclin E, and CDK4 protein levels in tumor tissues, n = 6. **(I, J)** Protein levels of E-cadherin, N-cadherin, and vimentin in tumor tissues from each group, n = 6. ns, *P* > 0.05; *, *P* < 0.05; **, *P* < 0.01; ***, *P* < 0.001.

Building on our previous findings that FBXL5 promotes YAP1 degradation and is post-transcriptionally upregulated by Gal-3. We observed that LGALS3-knockdown markedly promoted tumor growth, as indicated by increased tumor volume and weight compared with controls. In contrast, FBXL5-overexpression significantly suppressed tumor progression. Importantly, simultaneous LGALS3-knockdown and FBXL5-overexpression yielded intermediate tumor sizes, demonstrating that FBXL5 partially counteracts the pro-tumorigenic effect of LGALS3 loss (**Figures 6A–C**). There was no significant difference in body weight among the groups, suggesting that the genetic interventions did not cause obvious toxicity (**Figure 6D**). In tumor tissues, YAP1 protein levels were markedly increased in the shLGALS3 group but substantially reduced in the oeFBXL5 group, consistent with the *in vitro* observations (**Figures 6E, F**). FBXL5 effectively mitigated the pro-proliferative effect caused by LGALS3 loss by promoting YAP1 inhibition and its downstream cell cycle regulation and EMT phenotype (**Figures 6G–J**).

Overall, these findings substantiate that Gal-3 exerts a critical tumor-suppressive function *in vivo* through modulation of the FBXL5-YAP1 signaling axis.

## Discussion

The present study aimed to clarify the tumor cell–intrinsic role of Gal-3 in CRC and to define the mechanistic connection between Gal-3 and YAP1 regulation. By integrating multiple public cohorts and CRC single-cell transcriptomic atlases with *in vitro* functional assays and xenograft models, we found that LGALS3 expression is reduced in tumor tissues compared with non-tumor controls and is lower in malignant epithelial cells than in non-malignant epithelial populations. Mechanistically, we identified a Gal-3–FBXL5–YAP1 regulatory axis in which Gal-3 enhanced FBXL5-associated ubiquitination and promoted proteasome-dependent reduction of YAP1 protein abundance. Consistent with this mechanism, manipulation of LGALS3 or FBXL5 expression led to coordinated changes in YAP1 protein levels and tumor growth phenotypes, which were validated in both *in vitro* and vivo settings. Collectively, these results define a Gal-3–FBXL5–YAP1 axis that links Gal-3 to FBXL5-dependent control of YAP1 in CRC and provide mechanistic and experimental leads for evaluating its clinical relevance and translational potential; meanwhile, given that the *in vivo* validation was performed in xenograft models, its roles within an intact immune microenvironment remain to be further clarified in more immunologically relevant systems.

It was surprising to find a peculiar phenomenon during our initial exploration of public data, as *LGALS3* expression was found to decrease with CRC progression. This trend was consistently observed in both single-cell and bulk data (31, 32). Consequently, we also analyzed TCGA data and found that Gal-3 expression was indeed higher in the normal tissues of CRC patients compared to tumor tissues, a result we subsequently validated at both the gene and protein levels (**Figure 1**). In fact, there have been few studies in the past five years specifically addressing the exact mechanism of Gal-3 in colorectal cancer. Recent studies show that gleditsia saponin binds to LGALS3 and blocks ubiquitination-mediated proteasomal degradation at the Lys210 residue, thereby upregulating LGALS (15). These findings suggest that elevating LGALS3 levels exerts an anti-cancer effect. This is consistent with our results (**Figure 2**). Notably, clinical studies have reported elevated circulating Gal-3 levels in patients with CRC and an association with more advanced disease (33). At first glance, this appears inconsistent with the downregulation of LGALS3 observed in tumor tissues in our analyses. It is important to emphasize that serum Gal-3 more likely represents an integrated systemic readout contributed by multiple cellular sources within the tumor ecosystem. Prior studies have suggested that stromal compartments, including tumor-associated macrophages and cancer-associated fibroblasts, can secrete Gal-3 into the circulation in response to inflammatory cues (34–36). By contrast, bulk measurements at the tumor tissue level and tumor cell-focused analyses primarily reflect cancer cell-intrinsic programs. This provides a rationale for understanding the compartment-specific and potentially divergent roles of Gal-3 reported in the literature: extracellular Gal-3 has been described to act through microenvironmental and immune-associated mechanisms and may promote tumor progression in certain contexts, whereas intracellular Gal-3 in cancer cells can restrain growth, which is also supported by our data. Notably, our study primarily interrogates tumor cell–intrinsic programs, and direct causality on immune regulation will require validation in immunocompetent models and spatially resolved analyses. Collectively, our multi-omics analyses and functional experiments support a tumor cell-intrinsic Gal-3–FBXL5–YAP1 regulatory axis that limits CRC growth *in vitro* and in xenograft models. Meanwhile, the tissue-level cellular sources and spatial distribution of Gal-3 remain to be further clarified through systematic histologic and spatial mapping studies.

Subsequently, analyses of CRC scRNA-seq data together with *in vitro* validation supported a positive association between LGALS3 and FBXL5 expression. Moreover, elevated FBXL5 levels correlated with better patient survival, placing Gal-3 in a tumor-suppressive molecular context (**Figure 3**).Given that FBXL5 is a key constituent of the SCF E3 ubiquitin ligase complex, its coordinated expression with Gal-3 implies that Gal-3 may be involved in ubiquitin-dependent control of protein stability (37, 38). Therefore, the downregulation of Gal-3 in tumor tissues should not be interpreted as a simple loss of function but rather as a selective suppression adopted by cancer cells to evade its tumor-suppressive effects. This adaptive reduction further supports the critical inhibitory role of Gal-3 in the initiation and progression of CRC. Further mechanistic exploration revealed that Gal-3-overexpressing cancer cell subsets exhibited a robust response to the YAP1 signaling pathway. Given that YAP1 is also a key protein highly regulated by ubiquitination, we verified that FBXL5 can bind to YAP1 and modulate its expression through ubiquitination, thereby promoting YAP1 degradation (**Figure 4**, **5**). Finally, we validated this finding at the *in vivo* level using a human cell-derived xenograft mouse model. These results support that Gal-3 suppresses CRC growth through the FBXL5–YAP1 axis in tumor cell–intrinsic settings (**Figure 6**). Given the immunodeficient nature of the xenograft model, our *in vivo* data do not directly address immune regulation and should be interpreted accordingly.

The ubiquitination-mediated regulation of protein stability is a dynamic process occurring in both the cytoplasm and the nucleus (39, 40). Consistent with this multi-layer regulation, prior studies have highlighted that YAP1 activity is shaped not only by total protein abundance but also by the equilibrium of nucleocytoplasmic shuttling, which influences transcriptional output and clinical associations in CRC (41, 42). In line with these observations, cytoplasmic, rather than nuclear, localization of YAP1 has been associated with better overall and disease-free survival (43). However, in the current study we quantified YAP1 abundance and FBXL5-dependent ubiquitin–proteasome turnover but did not directly assess YAP1 subcellular localization dynamics by immunofluorescence or nuclear/cytoplasmic fractionation. Therefore, while altered turnover could plausibly affect the pool of YAP1 available for nuclear activity, this possibility remains to be experimentally validated. Taken together, our data support a tumor cell–intrinsic Gal-3–FBXL5 axis that promotes proteasome-dependent reduction of YAP1 protein levels in CRC models.

Gal-3 appears to exert tumor-suppressive effects in CRC in our datasets and experimental models, which contrasts with pro-tumoral roles reported in several other cancer contexts. Such discrepancies may reflect differences in tumor lineage, genetic background, and microenvironmental composition, as well as variations in experimental systems and readouts. Given the context-dependent nature of Gal-3 biology, future studies incorporating complementary cellular and animal models will be important to evaluate the generalizability of the Gal-3–FBXL5–YAP1 axis and to delineate potential model-specific effects. In addition, more detailed mapping of ubiquitination determinants, including the key residues and ubiquitin chain features governing YAP1 turnover, would further refine the mechanistic framework. Direct TEAD transcriptional output assays will also help refine pathway dependence. Moreover, although EMT-, apoptosis-, and cell-cycle-related protein changes were observed following LGALS3 manipulation, additional functional assays, including migration and invasion assays, apoptosis assays, and cell-cycle analysis, will be needed to further validate these phenotype-associated molecular changes. Finally, validation in larger and well-annotated patient cohorts, ideally incorporating immunofluorescence-based *in situ* mapping of Gal-3 distribution within tumor epithelial and stromal compartments together with clinical outcomes, will strengthen the translational relevance of our findings and may facilitate the development of Gal-3-related diagnostic and prognostic biomarkers. Looking forward, large online biobanks and public repositories, facilitated by user-friendly cohort extraction pipelines, will enable broader external validation and subgroup analyses of the Gal-3–FBXL5–YAP1 axis (24). In parallel, AI-driven multi-omics integration is expected to accelerate target prioritization and precision therapy design, supporting more forward-looking translational strategies (25, 26).

## Conclusions

Collectively, our findings suggest a tumor-suppressive function of Gal-3 in CRC and provide evidence compatible with a model in which Gal-3 promotes FBXL5-dependent ubiquitination and turnover of YAP1 to limit tumor progression. Future studies are warranted to delineate the precise molecular basis of this regulation, to connect it to defined YAP1-centered functional outputs, and to substantiate its clinical significance using independent patient cohorts and clinically annotated datasets. These investigations will be important for evaluating whether the Gal-3–FBXL5–YAP1 axis can be leveraged therapeutically in CRC.

## Data Availability

The original contributions presented in the study are included in the article/**Supplementary Material**. Further inquiries can be directed to the corresponding authors.
