# Supplementary material for: Galectin-3 promotes FBXL5-dependent ubiquitination and degradation of YAP1 to constrain colorectal cancer growth
Source: Front Immunol. 2026 Apr 21;17:1769656. doi: 10.3389/fimmu.2026.1769656 (PMC13139190; doi:10.3389/fimmu.2026.1769656)
Supplement: Supplementary file 2 [file DataSheet2.docx]

**Galectin-3 promotes FBXL5-dependent ubiquitination and degradation of YAP1 to constrain colorectal cancer growth**

Xiang Zeng^1, †^, TSZ KIN MAK^1, †^, Nan Li^2, †^, Jia Wang^1^, Zhiliang Huang^1,^ ^*^, Weiqun Lu^1^^,^ ^*^

^1^ Department of Gastrointestinal Tumor Surgery, Guangzhou Institute of Cancer Research, the Affiliated Cancer Hospital, Guangzhou Medical University, Guangzhou, China

^2^ Department of Internal Medicine, Guangzhou Institute of Cancer Research, the Affiliated Cancer Hospital, Guangzhou Medical University, Guangzhou, China

† These authors contributed equally to this work.

*Corresponding author

Weiqun Lu, PhD

Department of Gastrointestinal Tumor Surgery, Guangzhou Institute of Cancer Research, the Affiliated Cancer Hospital, Guangzhou Medical University, #78 Hengzhigang Road, Guangzhou, Guangdong 510095, China.

Email: [luweiqun_2020@163.com](mailto:luweiqun_2020@163.com)

**Supplementary Figures**


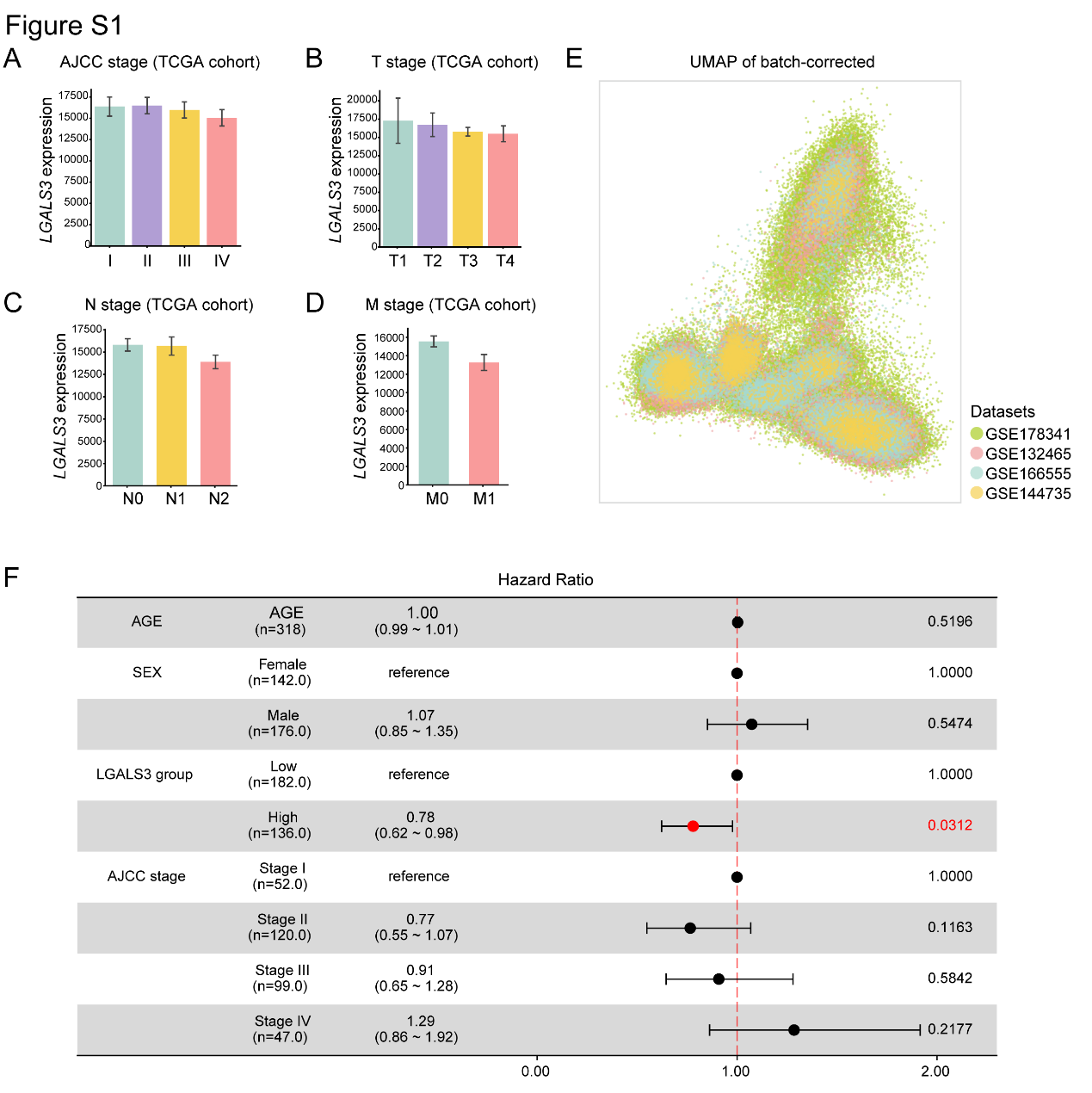


**Supplementary Figure 1 Public data mining.** (A-D) Expression of *LGALS3* across different AJCC stages, T, N, and M stages in the TCGA dataset. (E) Batch-corrected UMAP plot of single-cell sequencing data from four CRC patient datasets. (F) Multivariate Cox regression analysis of overall survival in the TCGA-CRC cohort identifies high *LGALS3* expression as an independent favorable prognostic factor.


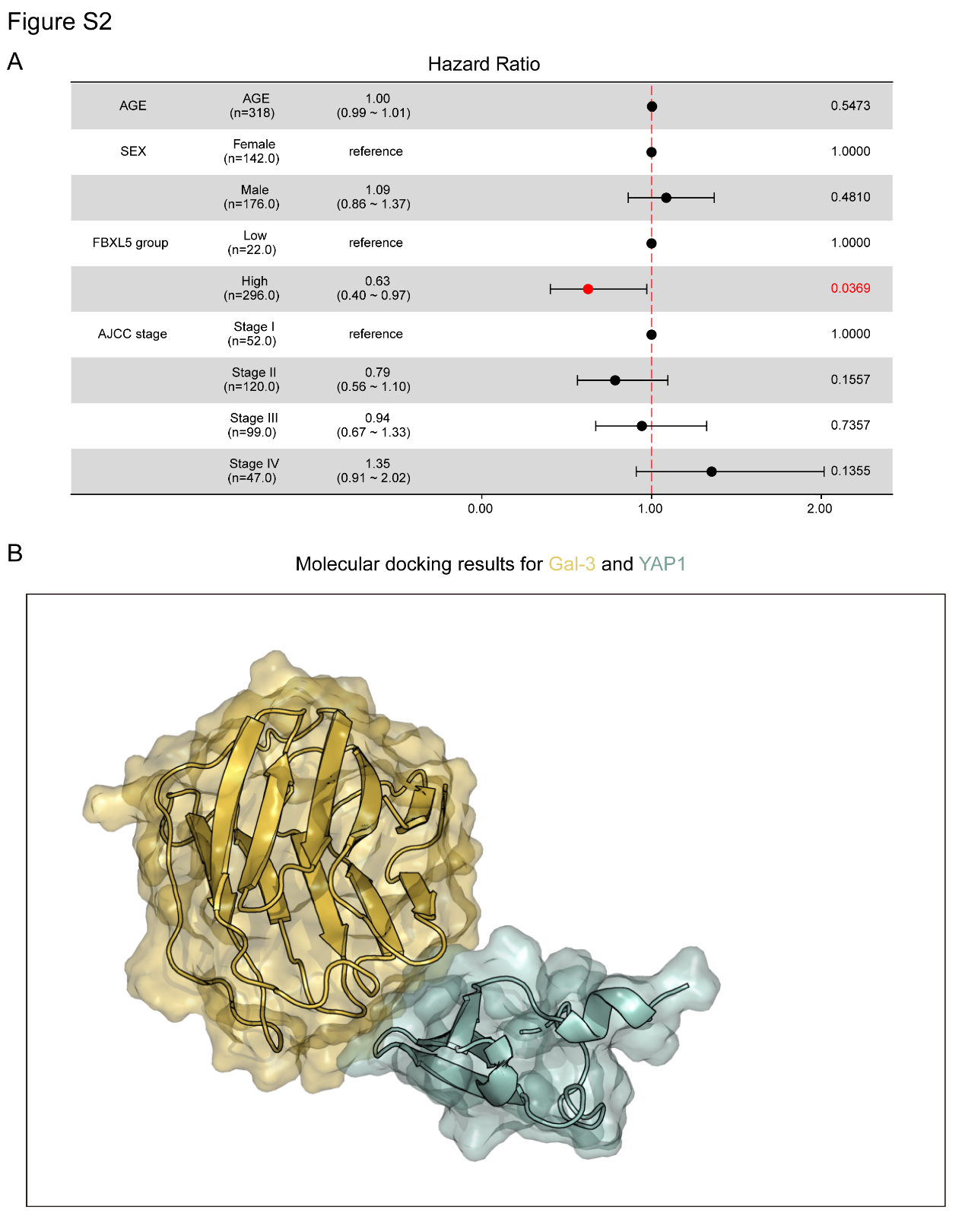


**Supplementary Figure 2 Clinical prognostic analysis and molecular docking results.** (A) Multivariate Cox regression analysis of overall survival in the TCGA-CRC cohort identifies high *FBXL5*expression as an independent favorable prognostic factor. (B) Molecular docking result of Galectin-3 and YAP1.
